# Traumatic Brain Injury during the SARS-CoV-2 Pandemics in Slovenia: A Single Center Study

**DOI:** 10.3390/jcm11237017

**Published:** 2022-11-28

**Authors:** Kevin Laufer, Karina Petek, Sofia Rakusa, Matej Rakusa, Martin Rakusa, Andrej Cretnik

**Affiliations:** 1 Faculty of Medicine, University of Maribor, Taborska 8, 2000 Maribor, Slovenia; 2 Traumatology Department, Divison of Surgery, University Medical Centre Maribor, Ljubljanska 5, 2000 Maribor, Slovenia; 3 Division of Neurology, University Medical Centre Maribor, Ljubljanska 5, 2000 Maribor, Slovenia; 4 Department of Endocrinology, Diabetes and Metabolic Disease, University Medical Centre Ljubljana, Zaloška 7, 1000 Ljubljana, Slovenia; 5 Faculty of Medicine, University of Ljubljana, Vrazov trg 2, 1000 Ljubljana, Slovenia

**Keywords:** traumatic brain injury, craniocerebral traumas, neurotrauma, clinical outcome, epidemiology, SARS-CoV-2 pandemic, Glasgow Coma Scale, Glasgow outcome scale extended

## Abstract

(1) Background: The SARS-CoV-2 pandemic had a significant impact on the management of traumatic brain injury (TBI). We aimed to compare the clinical characteristics and outcomes of TBI patients before and during the SARS-CoV-2 pandemic.; (2) Methods: We analyzed depicted data from existing medical records on sex, age, mechanism of injury, clinical performance at admission and discharge, neuroimaging, laboratory values at admission, mortality, duration of hospitalization, and referrals after discharge from the traumatology department for all adult patients during the SARS-CoV-2 pandemic and a year before. Variables were compared using the Chi-square or t-test between both groups.; (3) Results: Most patients had mild (n = 477), followed by moderate (11) and severe (11) TBI. Mild TBI was less frequent during the SARS-CoV-2 period (n = 174 vs. n = 303). The incidence of high falls increased during the SARS-CoV-2 period (14.5% vs. 24.7%; *p* < 0.05) in the group with mild TBI. Patients had similar mean Glasgow Coma Scales (GCS), Glasgow Outcome Scales-Extended (GOSE), and glucose levels at admission before and during the pandemic. Serum ethanol levels were significantly lower during the SARS-CoV-2 period (1.3 ± 0.7 mmol/L vs. 0.7 ± 1.2 mmol/L; *p* < 0.001). At discharge, the mean GCS was significantly lower (14.7 ± 1.8 vs. 14.1 ± 0.5; *p* < 0.05) for patients treated during the SARS-CoV-2 period than before the SARS-CoV-2 period. There were no differences in GOSE; (4) Conclusions: our results demonstrated a significant impact of SARS-CoV-2 pandemic on the frequency, mechanism, and consequences of TBI, and may help improve care for our patients.

## 1. Introduction

Traumatic brain injury (TBI) is an important medical condition that significantly impacts personal and public health. The overall incidence in Europe is estimated to be between 198 and 325 people per 100,000 per year, with fatal outcomes in 10.5 people per 100,000 per year. These numbers are even higher in the developing world. The direct and indirect costs of mild, moderate, and severe TBI are estimated in the USA at 90 billion dollars [1]. The economic evidence on TBI in Europe is scarce. Most of the data refer to hospitalized patients. Berg et al. compared costs between different European countries [2]. Although exact costs differed from country to country, the cost ratio between inpatients with concussions versus inpatients with severe brain injury was approximately 3 to 1 all over Europe. Overall, costs for patients with TBI are available only for some European countries. e.g., in the Netherlands, annual costs in 2012 were 314.6 million € [3], which is approximately 383.81 million € today [4].

TBI can be classified based on the mechanism (closed or penetrating head injury), location, or severity of the injury. The most common and simplest classification is based on the Glasgow Coma Scale assessed at admission, e.g., mild TBI (GCS > 13), moderate TBI (GCS between 9 and 12), and severe TBI (GCS < 9) [5]. Recently, the Brain Injury Special Interest Group Mild Traumatic Brain Injury (TBI) Task Force of the American Congress of Rehabilitation Medicine (ACRM) identified potential revisions for an update on the ACRM definition of mild TBI. Experts strongly agreed that updated diagnostic criteria for mild TBI should include levels of certainty, e.g., possible, probable, and definite. In addition, they should include neuroimaging evidence and specify alternative reasons for altered mental status. Therefore, rapid onset postconcussion symptoms would indicate at least a possible mild TBI [6].

Risk factors for TBI include age, sex, environmental factors such as humid or icy weather, motor vehicle accidents, falls, attacks, and alcohol consumption. After a TBI, it is not uncommon to observe altered glucose metabolism [7].

TBI can result in physical, cognitive, social, emotional, and behavioral symptoms. The most widely used scale for assessing global disability and recovery after TBI is the Glasgow Outcome Scale-Extended (GOSE). GOSE has eight categories, ranging from 1 “dead” to 8 “no problems” [8]. Although it is considered a gold standard and is used in clinical trials, it may not detect all TBI sequels. A recent study demonstrated that almost 20% of patients with a GOSE of 8/8 had impaired executive functions [9]. Therefore, more than one outcome measure should be used. Steinbuechel et al. explored psychometric characteristics of the patient-reported outcome measures in patients with TBI [10]. They found that the Generalised Anxiety Disorder 7 Item Scale, the Patient Health Questionnaire, the Posttraumatic Stress Disorder Checklist-5, the Rivermead Post-Concussion Symptoms Questionnaire, the Quality of Life after Brain Injury Scale, the Quality of Life after Brain Injury—Overall Scale, the 36-item Short Form Health Survey—Version 2, and the 12-Item Short Form Survey—Version 2 had medium to high correlations with the GOSE and GCS, and could be used as outcome measures in multiple domains.

At the moment, we do not have any specific treatment for TBI. Patients with moderate and severe TBI usually require complex treatment and rehabilitation by a multidisciplinary professional team [11]. However, patients with mild TBI may have post-concussive symptoms. Therefore, we need a prognostic model to plan the early rehabilitation of patients with TBI. Recently, Mikolic et al. compared several different prognostic models. Their analysis demonstrated that there is no ideal predictive model. Parameters which may improve prediction were TBI-related, and psychological symptoms collected two weeks after TBI improved predictions [12]. In the future, we need prediction models that will include clinical outcomes and biomarkers.

Outcomes can range from complete recovery to permanent disability or death. Even a mild TBI may break neuronal integrity, change brain metabolism, and increase cell membrane turnover, which may cause long-term neurodegeneration [13]. Therefore, we should actively seek possible sequelae with psychological evaluation [10] or potential radiological biomarkers, such as N-acetyl-aspartate, glutamate, and choline [13].

Patients with mild TBI generally have good outcomes, while those with moderate or severe TBI may be permanently impaired or even die. However, up to 50% of uncomplicated TBI patients with a GOSE of 8/8 do not fully recover at 12 months after mild TBI without visible injuries in brain MRI. Moreover, around 1% of initially mild TBI patients die from the so-called mild TBI [13]. A recent study also demonstrated that craniectomy might improve a patient’s quality of life in the long term despite not improving physical outcomes six months after neurotrauma [14]. In the majority of patients, TBI does not have a great impact on their quality of life. However, one-third of the patients have neuropsychiatric sequelae, which significantly reduce their quality of life [15]. Neuropsychiatric impairments are most significant in older females with moderate or severe TBI; it may take a decade before they fully develop [16,17].

### Epidemiology and Management of TBI during the SARS-CoV-2 Pandemic

The SARS-CoV-2 pandemic has brought unique challenges in everyday life. In Slovenia, during the SARS-CoV-2 pandemic, the government imposed three lockdowns. The first lockdown was from 12 March to 31 May 2020. The second lockdown was from 27 October 2020 to 8 February 2021, except for one week during the Christmas holidays. The last lockdown was short, and lasted ten days in April 2021.

Most restaurants, as well as all bars and nightclubs, were closed during the lockdowns. Only business customers were allowed to visit restaurants during lunchtime. People were also denied free movement through the country for a shorter period with some exemptions. On the other hand, in the period between lockdowns, all restaurants were working normally, and there was no ban on alcohol. Despite prevention methods, the number of infected patients with SARS-CoV-2 increased. The prevalence of confirmed SARS-CoV-2 cases from March 2020 to December 2020 was 105,906 patients [18].

Changes in daily life were also visible in the epidemiology of TBI. Retrograde analysis from single-centers in Austria [19], Italy [20], France [21], Finland [22], the Netherlands [23], Ireland [24], and a multi-center study from Austria, the Czech Republic, and Switzerland [25], as well as studies around the world [26,27], demonstrated a decreased number of TBI cases in comparison with previous years and months preceding the pandemic. In addition, the mechanism of the injuries, patterns, severity, and outcomes were also influenced by the SARS-CoV-2 pandemic.

A recent meta-analysis demonstrated that low- and middle-income countries were most affected, where mortality rates increased due to TBI [28]. In some countries, road accidents paradoxically increased despite lockdowns [29], while others decreased [27]. Several countries also recorded more assaults [27,29].

Only a few studies have analyzed TBI epidemiology and medical features in central Europe during the SARS-CoV-2 pandemic. Despite significant changes worldwide, there was no report from Slovenia, which may add additional information on TBI management in central Europe. Therefore, our study aimed to analyze the impact of the SARS-CoV-2 pandemic on the common risk factors and management of patients treated due to TBI.

## 2. Materials and Methods

### 2.1. Population

We conducted a single-center, retrospective, observational study at the department of traumatology in the University Medical Center of Maribor in the Styria region of Slovenia. In addition, patients with moderate or severe TBI from two neighboring regions also migrated to our department. The region has a population of 325,994 and, with neighboring regions, approximately 500,000 inhabitants in total, which represents 25% of the Slovenian population. Most people live in an urban area. A minority of the population live in rural area [30]. The region has a continental climate, with an average air temperature of between −0.1 °C in January to 21 °C in July, and it is well known for its vines [31]. There is no data on alcohol consumption in our region. However, in 2020, people 15 years or older drank the equivalent of 9.8 liters of pure alcohol on the national level [32].

The first case of SARS-CoV-2 in Slovenia was documented on 4 March 2020 [18]. Therefore, we included all patients treated between March and December 2020 in a “SARS-CoV-2 period” group. As previously mentioned, this period also included 20 weeks of lockdown. For the control group, we used patients treated between March and December 2019.

### 2.2. Study Design and Scales

Inclusion criteria were the diagnosis of traumatic brain or head injury or polytrauma at admission, as was similar to previous studies on the SARS-CoV-2 pandemic’s impact on TBI epidemiology [23,33]. We analyzed depicted data from existing medical records on risk factors (sex, age, mechanism of injury) and management (clinical performance at admission and discharge, neuroimaging, mortality, duration of hospitalization, and referrals after discharge from the traumatology department for all adult patients 18 years or older). Alcohol consumption is a known risk factor for TBI. In addition, hypoglycemia may also lead to falls, which is why we also examined laboratory values for those parameters at admission.

The patient’s performance was evaluated with the GCS at admission and at discharge and with the GOSE.

The Institutional Review Board of the University Medical Center of Maribor in Slovenia approved the study (approval number UKC-MB-KME-41/22).

### 2.3. Statistical Analysis

We performed descriptive analysis for each period for the demographic features of the patients (age and sex), the most prevalent mechanisms and consequences of the injuries, the medical state of patients at admission and discharge (GCS, GOSE), serum laboratory values at admission (glucose, ethanol), and the length of hospitalization.

After we performed descriptive analysis, we noted that most of the patients in our sample had mild TBI. There were only a few patients with moderate or severe TBI. In order to avoid a mixed sample, we compared differences between the two time periods for the mild TBI only, using either the Chi-square or *t*-test, and *p* values < 0.05 were considered significant.

## 3. Results

We included 498 patients in our study. More than 67% of patients were men. Most patients had mild TBI (n = 477 patients; 95%; Table 1), followed by moderate TBI (n = 11 patients; Table 2) and severe TBI (n = 11 patients; Table 3). Due to the small sample size in the moderate and severe TBI groups, we did not make any further comparisons.

The most common mechanism of injury was falling from the same level, followed by traffic accident, and fall from height for patients with mild TBI (Table 1). There were similar results for patients with moderate or severe TBI (Table 2 and Table 3). Twenty-four patients in the pre-SARS-CoV-2 period and eighteen during SARS-CoV-2 had more than one type of injury mechanism (Table 1 and Table 3).

### Differences in Epidemiology and Outcome for Patients with Mild TBI before and during the SARS-CoV-2 Period

Patients with mild TBI fell more frequently from height during the SARS-CoV-2 period than before SARS-CoV-2 (Table 4). However, other types of injury did not differ between periods.

Mean GCS and GOSE were similar in both groups at the admission before and during the SARS-CoV-2 period (Table 4). However, significant differences were found for the GCS at discharge. Patients during the SARS-CoV-2 period had significantly lower GCS than those in the pre- SARS-CoV-2 period (Table 4). No significant differences were found for the GOSE at discharge. Patients spent approximately ten days at the hospital during both periods.

A significant difference was found between serum ethanol levels between periods. Patients had significantly lower levels of ethanol in serum during the SARS-CoV-2 period than in the control group. Serum glucose levels were mildly elevated and did not differ between groups (Table 4).

## 4. Discussion

The present study demonstrated that the COVID-19 pandemic significantly impacted TBI patients. Furthermore, we found differences in frequency, mechanism, and consequences between patients treated before and during the COVID-19 period.

Our results demonstrate that the number of patients with mild TBI declined by 43% during the SARS-CoV-2 pandemic (Table 1). Similar observations were reported across Europe and around the world. For example, in Tyrol, Austria, where they had strict lockdowns and curfews, the frequency of TBI declined by 46% [19], in Italy by 49% [20], and in the USA by 48.5% [29].

However, the frequency of moderate or severe TBI incidence before and during the SARS-CoV-2 period differs among studies. For example, Grassner et al. reported in a large international study that patients who needed neurosurgical intervention from Austrian, Swiss, and Czech centers were fewer during the SARS-CoV-2 pandemic than in previous years [25]. On the other hand, a recent meta-analysis did not find significant differences in frequencies for moderate and severe TBI before and during the SARS-CoV-2 pandemic [28]. Our results follow the results of the meta-analysis. We did not find any significant differences, although our sample for patients with moderate and severe TBI was much smaller than for mild TBI.

Overall, we did not find any differences between males and females in mild TBI. However, we found a significant increase in high falls during the SARS-CoV-2 period than for the pre-SARS-CoV-2 period. Increased high fall frequency was probably not due to suicide attempts. The number of suicides in Slovenia declined during the SARS-CoV-2 pandemic [34]. A possible reason for the increase could be that construction workers were allowed to continue working despite the lockdown.

Changes in the mechanisms of TBI differed between countries and most probably reflect socio-economical status. E.g., in Austria, they did not treat any skiing-related TBI injury during the lockdown and had a significantly lower frequency of skull fractures [19], while in New York, USA, they had more patients with TBI due to road traffic accidents [29]. On the other hand, in Italy, they did not find any differences in mechanisms leading to the TBI and concomitant lesions [20]. In a recent meta-analysis, mechanisms differed between low- and high-income countries, and assaults were a more frequent cause of TBI during the SARS-CoV-2 period [25].

In addition, we found significant differences in ethanol serum levels and no significant differences in glucose levels (Table 4). Less alcohol consumption due to lockdown, decreased mobility, and fewer social events may also have contributed to the different mechanisms and the decline in TBI. Consumption of alcohol per person (15 years or older) was lower compared to 2019 and 2021 by the equivalent of pure alcohol by 1.25 L and 0.82 L, respectively [32]. Our findings concurred with a report from Rajalu et al. [35] which found that decreased sales of alcoholic beverages during the SARS-CoV-2 pandemic were associated with reduced TBI.

The pandemic has influenced the outcome of TBI in several countries (Table 5). The authors focused on the frequency and severity of TBI, but none of the mentioned studies reported GOSE scores. Therefore, we can only partly compare clinical outcomes with our study. Our patients had slightly but significantly lower GCS at discharge during the SARS-CoV-2 period. Therefore, their performance was worse in comparison to the pre- SARS-CoV-2 period. However, we did not observe this effect on the GOSE, which is more sensitive than GCS. On average, patients with mild TBI in both periods were moderately disabled (Table 4).

Similar reports were found around the world. Differences were especially evident for the patients with major trauma. Several centers reported a higher mortality rate during the SARS-CoV-2 pandemic than before it [36]. One of the reasons could be changes in the protocols. For example, in the Netherlands, fewer patients with mild to moderate TBI were referred to the ICU during the SARS-CoV-2 pandemic than before it [36]. On the other hand, the mortality rate remained the same in Finland, where they did not change protocols for referrals to the ICU [22]. The major change in protocol in Slovenia was observed in rehabilitation. Although differences were not significant between observed time periods, ten times fewer patients were referred to the rehabilitation facilities immediately after discharge during the SARS-CoV-2 period. One reason was that those facilities did not accept referrals due to strict anti-COVID measures and lockdowns. It is also worth noting that most of the patients had neuro-rehabilitation during the hospitalization at the department of traumatology, and they continued with it in the community health care center or began with rehabilitation after a certain period after discharge.

The major strength of our study is the large sample size. The analyzed data was from the relatively long SARS-CoV-2 period, which included lockdowns and relatively mild restrictions. In comparison to previous studies, we reported data from the longer lockdown period (Table 5). In addition, we analyzed other risk factors for TBI, such as ethanol and glucose serum levels, and reported clinical outcomes for mild, moderate, and severe TBI.

However, the present study also had some limitations. We performed a single-center, retrospective study. Our center covers approximately 16% of the Slovenian population with mild TBI and 25% with moderate and severe TBI. Therefore, we may not have fully expanded our results to the general population. We may also not entirely have excluded that some cases of mild TBI were treated at the two other local hospitals. On the other hand, all moderate and severe TBI were referred to our institution. We also may have missed factoring in patients who had COVID-19 and TBI simultaneously because they were referred to the COVID-19 department.

## 5. Conclusions

The SARS-CoV-2 pandemic had a significant impact on the epidemiology of neurotrauma. The severity, mechanisms, and outcomes of TBI differ among countries. However, those factors also reflect social factors restricted during the lockdown periods, changes in lifestyle, and altered patient care protocols. In order to better prepare for the future of pandemics, it is important to understand this and to improve our care for patients with TBI.

## Figures and Tables

**Table 1:** Epidemiology and management of patients with mild TBI.

		Pre-SARS-CoV-2 Period N = 303		SARS-CoV-2 Period N = 174	
Epidemiology/Risk factors	Men	209	(69%)	110	(63.2%)
	CT	230	(75.9%)	130	(74.7%)
	MRI	1	(0.3%)	0	(0%)
	Skull fracture	29	(9.6%)	24	(13.8%)
	Fall from height *	44	(14.5%)	43	(24.7%)
	Fall from the same level	176	(58.1%)	89	(51.1%)
	Penetration injury	3	(1%)	0	(0%)
	Polytrauma	7	(2.3%)	10	(5.7%)
	Traffic accident	58	(19.1%)	40	(23%)
	Head blow	17	(5.6%)	11	(6.3%)
	Fight	19	(6.3%)	5	(2.9%)
	More than one mechanism of injury	24	(7.9%)	17	(9.8%)
Management after TBI	Referral to the rehabilitation center at discharge	10	(3.3%)	1	(0.6%)

TBI—traumatic brain injury, CT—computer tomography, MRI—magnetic resonance imaging, *—*p* < 0.05.

**Table 2:** Epidemiology and management of patients with moderate TBI.

		Pre-SARS-CoV-2 Period N = 7		SARS-CoV-2 Period N = 4	
Epidemiology/Risk factors	Men	5	(71.4%)	2	(50%)
	Age	56 years	10.7 years	52.5 years	22.1 years
	CT	6	(85.7%)	4	(100%)
	MRI	0	(0%)	0	(0%)
	Skull fracture	0	(0%)	1	(25%)
	Fall from height	1	(14.3%)	1	(25%)
	Fall from the same level	5	(71.4%)	1	(25%)
	Penetration injury	0	(0%)	0	(0%)
	Polytrauma	1	(14.3%)	1	(25%)
	Traffic accident	1	(14.3%)	2	(50%)
	Head blow	0	(0%)	0	(0%)
	Fight	0	(0)	0	(0)
	More than one mechanism of injury	0	(0%)	0	(0%)
Management after TBI	Referral to the rehabilitation center at discharge	0	(0%)	0	(0%)

TBI—traumatic brain injury, CT—computer tomography, MRI—magnetic resonance imaging.

**Table 3:** Epidemiology and management of patients with severe TBI.

		Pre-SARS-CoV-2 Period N = 4		SARS-CoV-2 Period N = 7	
Epidemiology/Risk factors	Men	3	(75%)	7	(100%)
	Age	55.3 years	17.5 years	50.9 years	19.6 years
	CT	4	(100%)	6	(85.7%)
	MRI	0	(0%)	0	(0%)
	Skull fracture	0	(0%)	4	(57.1%)
	Fall from height	0	(0%)	0	(0%)
	Fall from the same level	3	(75%)	2	(28.6%)
	Penetration injury	0	(0%)	0	(0%)
	Polytrauma	0	(0%)	4	(57.1%)
	Traffic accident	0	(0%)	5	(71.4%)
	Head blow	0	(0%)	1	(14.3%)
	Fight	1	(25%)	0	(0%)
	More than one mechanism of injury	0	(0%)	1	(14.3%)
Management after TBI	Referral to the rehabilitation center at discharge	0	(0%)	0	(0%)

TBI—traumatic brain injury, CT—computer tomography, MRI—magnetic resonance imaging.

**Table 4:** Clinical characteristics and outcomes of mild TBI patients during pre- SARS-CoV-2 and SARS-CoV-2 periods.

	Pre-SARS-CoV-2 Period N = 303				SARS-CoV-2 Period N = 174						
	Mean	Std. Deviation	95% Confidence Interval for Mean		Mean	Std. Deviation	95% Confidence Interval for Mean				
			Upper	Lower			Upper	Lower	*t*	df	*p*
Age (years)	61.9	19.8	64.1	58.9	59.7	25.0	63.4	56.0	−1.6	475	0.111
Number of days in the hospital	9.5	16.2	11.3	7.7	7.3	8.3	8.5	6.1	1.9	475	0.093
GCS at admission	14.8	0.5	14.9	14.7	14.8	0.5	15.0	14.8	−1.2	475	0.246
GCS at discharge	14.7	1.8	14.9	14.5	14.1	0.5	14.1	13.9	2.4	460	0.018
GOSE at admission	7.8	0.8	7.9	7.7	7.8	0.8	7.9	7.7	0.05	463	0.957
GOSE at discharge	6.5	1.7	6.7	6.3	6.4	2.0	6.7	6.1	0.55	460	0.586
Serum glucose level at admission (mmol/L)	7	3.9	7.5	6.5	7.3	2.9	7.8	6.8	−0.75	396	0.452
Serum ethanol level at admission (mmol/L)	1.3	0.7	1.4	1.2	0.7	1.2	0.9	0.5	3.7	401	<0.001

GCS—Glasgow Coma Scale, GOSE—Glasgow Outcome Scale-Extended.

**Table 5:** Epidemiology of TBI in Central Europe.

Country	Lockdown Period [Weeks]	Number of Patients [n]	Mild TBI [n (%)]	Moderate TBI [n (%)]	Severe TBI [n (%)]	Mortality Quarantine Period [n (%)]
Slovenia	20	177	159 (89.8)	11 (6.2)	7 (4.0)	0 (0)
Austria (Tyrol) [19]	3	13	9 (69)	0 (0)	4 (31)	2 (15)
Italy [20]	8	26	– *	– *	– *	– *
France [21]	11	6	– *	– *	– *	0 (0)
Finland [22]	11	67	– *	– *	– *	0.85 (CI 0.76–0.94) ^‡^
The Nederlands [23]	3	91	90 (98.9)	1 (0.7)	0 (0)	– *
Ireland [24]	13	32	8 (24.1) ^†^	10 (31.0) ^†^	14 (44.8) ^†^	5 (15.6) •
Austria [25]	4.3	27	– *	– *	– *	2 (7.7) •
Czechia [25]	4.3	48	– *	– *	– *	2 (4.3) •
Switzerland [25]	4.3	18	– *	– *	– *	3 (16.7) •

TBI—traumatic brain injury; n—number of patients; *—no data provided from authors; ^‡^—mortality presented as AUC of the predicted risk of death model; ^†^—assessed with GCS; •—30-day mortality.

## Data Availability

Due to legal restrictions, data can not be shared freely.
